# Injuries of the isolated larynx-hyoid complex in post-mortem computed tomography (PMCT) and post-mortem fine preparation (PMFP) - a comparison of 54 forensic cases

**DOI:** 10.1007/s00330-020-06770-4

**Published:** 2020-03-31

**Authors:** Karla Maria Treitl, Laura Isabel Aigner, Evgenij Gazov, Florian Fischer, Regina Schinner, Christine Schmid-Tannwald, Sonja Kirchhoff, Michael Karl Scherr

**Affiliations:** 1 Department of Radiology, University Hospital, LMU Munich, Pettenkoferstr. 8a, 80336 Munich, Germany; 2 Department of Radiology, Klinikum Kempten, Kempten, Germany; 3 Department of Radiology, BG Unfallklinik Murnau, Murnau am Staffelsee, Germany; 4 Institute of Forensic Medicine, Ludwig Maximilians University, Munich, Germany

**Keywords:** Autopsy, Multidetector computed tomography, Neck injuries, Larynx

## Abstract

**Objectives:**

To assess the diagnostic accuracy (ACC) of post-mortem computed tomography (PMCT) for fractures of the isolated larynx-hyoid complex (LHC) in comparison to post-mortem fine preparation (PMFP).

**Methods:**

This monocentric prospective study enclosed 54 LHCs that were extracted during autopsy, fixed in formalin, and underwent a PMCT scan (64-row multidetector CT, helical pitch). Two radiologists independently analyzed the LHC scans for image quality (IQ) and fractures (4-point Likert scales). A specialized forensic preparator dissected the specimens under the stereomicroscope. The PMFP results were standardized documented, and used as the standard of reference for the comparison to PMCT.

**Results:**

The PMCT-IQ of 95% of the LHC images was rated as good or excellent. IQ was decreased by decay, incisions during autopsy, and separation of the hyoid from the cartilaginous components in 7, 3, and 12 specimens, respectively. PMFP detected 119 fractures in 34 LHCs (63.0%). PMCT identified 91 fractures in 32 specimens (59.3%). PMFP and PMCT significantly agreed concerning the location (Cohen’s *κ* = 0.762; *p* < 0.001) and the degree of dislocation (*κ* = 0.689; *p* < 0.001) of the fractures. Comparing PMCT to PMFP resulted in a sensitivity of 88.2%, a specificity of 90.0%, and an ACC of 88.9% for the LHC. The ACCs for the hyoid, thyroid, and cricoid were 94.4%, 87.0%, and 81.5%, respectively. PMCT procedure was significantly faster than PMFP (28.9 ± 4.1 min vs. 208.2 ± 32.5 min; *p* < 0,001).

**Conclusions:**

PMCT can detect distinct injuries of the isolated LHC and may promptly confirm violence against the neck as cause of death. PMFP outmatches PMCT in the detection of decent injuries like tears of the cricoid cartilage.

**Key Points:**

*• Post-mortem computed tomography is able to assess fractures of the larynx-hyoid complex.*

*• Prospective monocentric in vitro study showed that post-mortem computed tomography of the larynx-hyoid complex is faster than post-mortem fine preparation.*

*• Post-mortem computed tomography can confirm violence against the neck as cause of death.*

## Introduction

The first application of CT in a forensic context was for the examination of cranial gunshot wounds in 1977 [1]. Nowadays, post-mortem computed tomography (PMCT) is discussed as an alternative to autopsy [2–4] or has become a routine procedure before autopsy [5]. PMCT is highly efficient in the evaluation of traumatic fractures and gunshot wounds as well as intracranial injuries [5–7]. Decay and post-mortem changes of the cardiovascular system impair the image analysis [4].

Considering this, PMCT seems to be a promising tool in forensic cases of violent neck injuries, as these are frequently associated with fractures of the larynx-hyoid complex (LHC) [8]. Hanging, manual, and ligature strangulation are the most frequent causes of violent neck injuries. Traffic accidents, window falls, and assaults are other causes of LHC fractures [8, 9]. Objective verification of LHC fractures via PMCT is essential in decomposed bodies and is advantageous in forensic cases, when petechial hemorrhage or facial congestion is missing.

To date, only a few studies have compared the detectability of LHC fractures by PMCT to that by autopsy [10–13]. Moreover, no studies have compared the results of PMCT to specific post-mortem fine preparation (PMFP) of the larynx performed under a stereomicroscope. Thus, the aim of this study was to assess the diagnostic accuracy of PMCT for fractures of the isolated LHC, preserved in 2% formaldehyde solution, and compare it to that of PMFP as diagnostic gold standard.

## Materials and methods

### Specimens

The study was designed as a prospective monocentric in vitro cohort study comparing PMCT to PMFP as the current gold standard. The study protocol followed the principles of the Declaration of Helsinki and was approved by the institutional ethical review board (proposal no. 151/08; date of approval: February 12, 2008).

Specimens were consecutively recruited from autopsies between July 2013 and May 2014 according to the following criteria: (1) forensic cases with an inconclusive result of the autopsy regarding the determination of the cause of death, (2) forensic cases with suspicion of violent neck injuries, and (3) forensic cases older than 15 years at the time of death. Forensic cases with severe destructive changes that did not allow an assessment under the stereomicroscope and cases with a distinct, not-neck-related cause of death proven by autopsy were excluded. After the completion of the criminal investigation and the finalization of all forensic examinations, the distinct causes of death were determined and recorded.

### PMCT

LHCs were extracted during autopsy and preserved in plastic containers (10 × 10 × 12 cm^3^) filled with 2% formaldehyde solution. The containers were fixed with tape to the table of a 64-row multidetector CT scanner (Optima CT 660, GE Healthcare Systems) and underwent a helical scan with a slice thickness of 0.625 (120 kV, 300 mA) at a recording speed of 10.62 mm/s and a pitch factor of 0.05 to prevent motion artifacts of the specimens within the fluid. Scan time, volume-weighted CT dose index (CTDIvol, mGy), and dose length product (DLP, mGy/cm) were recorded.

Multiplan reformations were calculated with a slice thickness of 0.7 mm using the “standard-soft-tissue” and the “bone-plus” kernel settings of the CT scanner’s workstation. Using the OsiriX Lite software (32-bit, version 5.8; Pixmeo SARL), the PMCT images were orthogradly adjusted and analyzed on an iMac (27 in., Mid 2011, OS X 10.9.3). The duration of the post-processing was recorded.

### Image analysis

Two radiologists with 6 years (K.T.) and 12 years (M.S.) of experience, who were blinded to the PMFP results, independently analyzed the image quality (IQ) of the hyoid, the thyroid, and the cricoid cartilage on a 4-point Likert scale [14]: 1 = poor, definite diagnosis impossible; 2 = fair, evaluation of major findings possible; 3 = good, definite diagnosis possible; and 4 = excellent, exact diagnosis possible. Any separation of the hyoid from the remaining components and any advanced decay indicated by multiple bubbles of entrapped air were documented.

The LHC components were divided into 5 subareas of the hyoid (corpus, rostral, and occipital parts of the right horn, rostral and occipital parts of the left horn), 7 subareas of the thyroid (centerline, left and right shields, upper and lower right horns, upper and lower left horns), and 4 subareas of the cricoid (rostral and occipital centerlines, right and left sides). The degree of dislocation was documented for each fracture in each subarea on a 4-point Likert scale [14]: 0 = no fracture, 1 = incomplete fracture (fissure), 2 = non-displaced fracture, and 3 = displaced fracture. The windowing that was used to find a definite diagnosis (bone or soft tissue window or both) and common normal variants (foramen thyroideum, cartilago triticea, asymmetry/agenesis/aplasia of a thyroidal or hyoidal horn, Eagle syndrome) were documented [15, 16].

Images were reviewed after 2 weeks, if the readers disagreed; a consensus decision was made; and the results were adapted. The time required for image analysis was recorded.

### PMFP

During autopsy, LHCs were separated from the respiratory system by an incision right above the hyoid bone and an incision 4 cm below the cricoid. The specimens were kept in 2% formaldehyde solution for at least 24 h to prevent post-mortem hemorrhage.

**Fig. 1:**
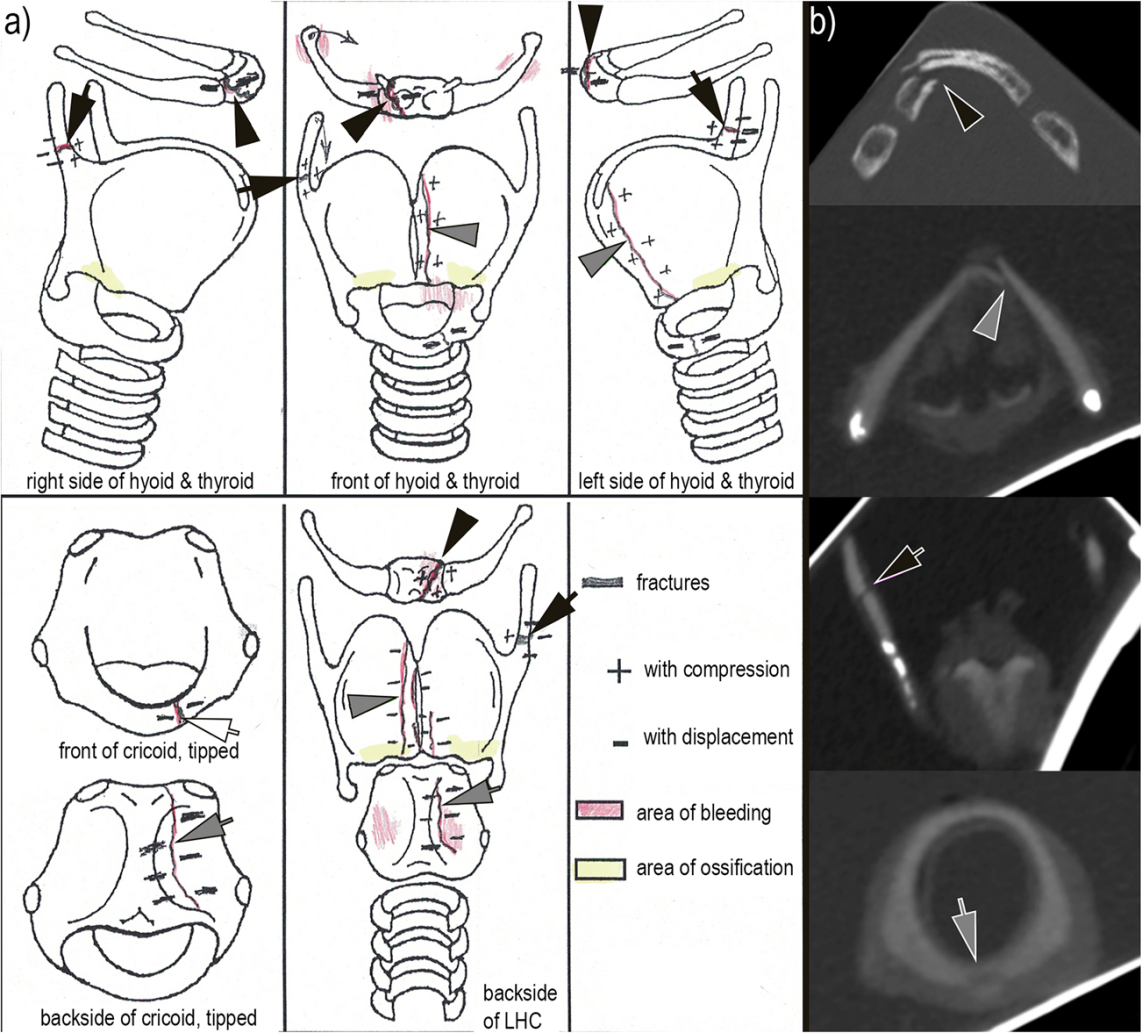
Comparison of PMFP and PMCT of the isolated LHC. **a** The standardized documentation of PMFP (modified according to the scheme of Korjakina and Mishin [17]) marks a displaced, right-sided fracture of the corpus of the hyoid (black arrowheads); a non-displaced fracture of the right-sided upper horn of the thyroid (black arrows); a displaced fracture of the left shield of the thyroid, which is close to the centerline (gray arrowheads); and a para-central fissure of the occipital (gray arrows) and rostral (white arrow) cricoid. **b** The PMCT slices reformatted in the bone window show the fracture of the corpus of the hyoid (black arrowhead in the axial view), the fractures of the left shield (gray arrowhead in the axial view) and the right-sided upper horn of the thyroid (black arrow in the coronal view), and the para-central fissure of the occipital cricoid (gray arrow in the axial view); the fissure of the rostral cricoid cannot be detected in PMCT

**Fig. 2:**
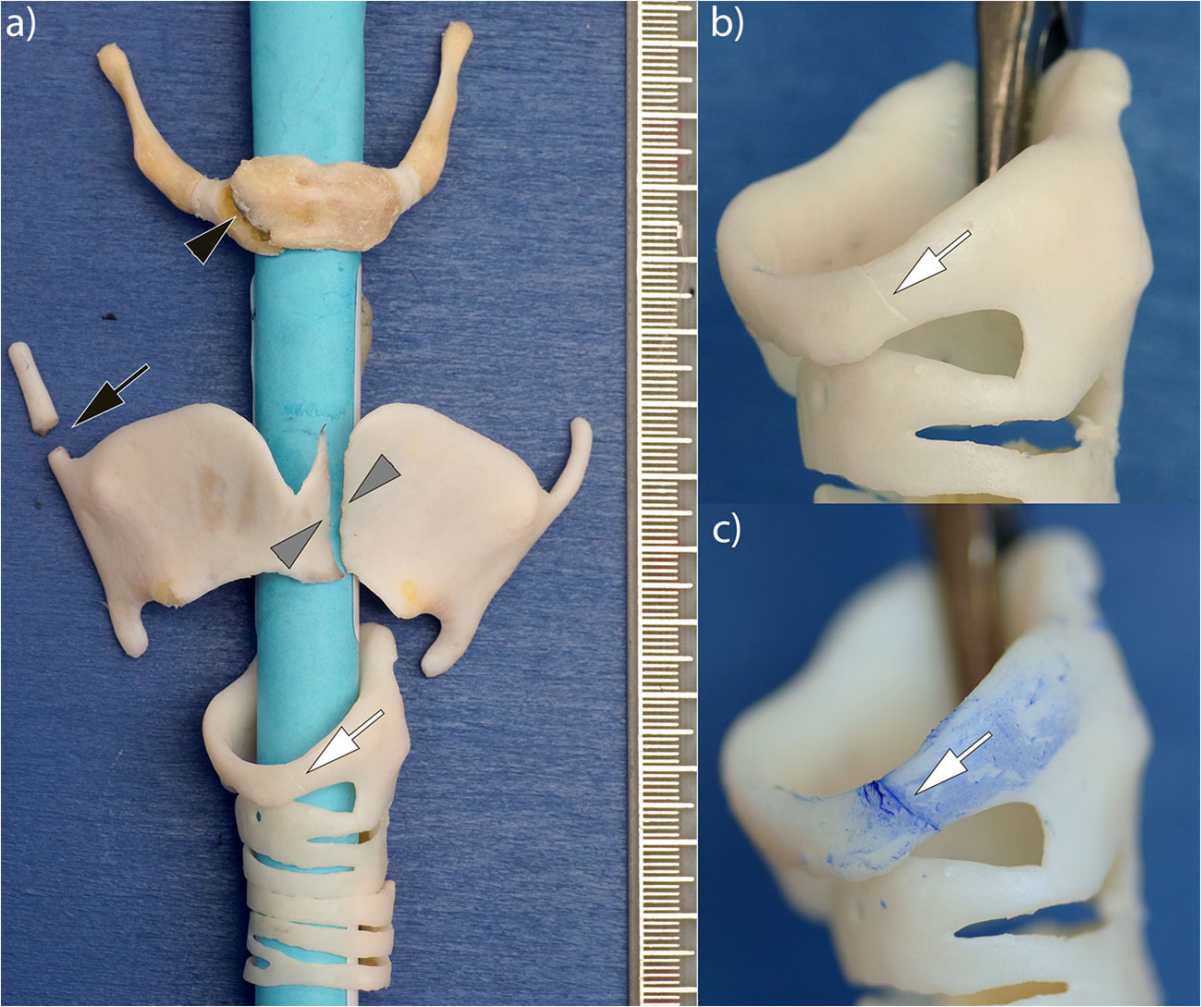
Photographs of the isolated LHC specimen from Fig. 1 after the PMFP. **a** Anterior overview of the LCH specimen with a right-sided fracture of the corpus of the hyoid (black arrowhead), a fracture of the right-sided upper horn of the thyroid (black arrow), a para-central fracture of the left shield of the thyroid (gray arrowheads), and a barely visible fissure of the left para-central rostral cricoid (white arrow). **b** Magnification of the cricoid: the fissure is still barely detectable (white arrow). **c** Magnification of the cricoid after the application of aniline dye: the entire dimension of the fissure is clearly visible (white arrow)

A specialist in PMFP of the LHC with more than 20 years of experience performed the PMFP according to Korjakina and Mishin [17]. The LHC components were examined for fractures and bleeding indicating fractures using a stereomicroscope. Small fissures were visualized using blue ink. Perturbing soft tissue was carefully removed. The location of the LHC fractures and their degrees of separation were recorded on a standardized documentation sheet (Figs. 1 and 2). The duration of the PMFP was recorded as well.

### Statistics

Statistical analysis was performed with IBM SPSS Statistics 22 (IBM Corporation) and SAS (version 9.4) for Windows (SAS Institute, Inc.).

Descriptive statistics of the study cohort were reported as follows: means and standard deviations for continuous variables, and frequencies and percentages for categorical variables. The chi-square test was applied to compare image quality and windowing of the three LHC components in PMCT. The inter-reader agreement was calculated using Cohen’s kappa value (*κ*). Cohen’s kappa value was also used to compare the results of PMCT and PMFP regarding the localization and the degree of dislocation of the fractures. Sensitivity, specificity, and accuracy were calculated with a standard 2 × 2 contingency table. *P* values below 0.05 were considered significant.

## Results

### Specimens

A total of 54 LHCs were examined in PMCT and PMFP. Of the 58 investigated bodies, one specimen was excluded because at the time of death, the age was under 15 years. Another one was excluded due to burns covering two thirds of its surface. Two LHCs were excluded because the majority of both specimens were destroyed by decay. The remaining 54 specimens were extracted from 21 female and 33 male bodies (38.9% and 61.1%) with a mean age of 53.0 ± 21.2 years at the time of death, with ages ranging from 15 to 95. Table 1 presents the causes of death and anthropometric data of the investigated bodies.

**Table 1:** Anthropometric data and causes of death of the investigated bodies (*n* = 54); the italic values represent the total amount of cases

Anthropometric data	
Male sex	33/61.1%
Age (years)	53.0 ± 21.2
Weight (kg)	72.5 ± 15.2
Height (m)	1.7 ± 0.1
BMI (kg/m^2^)	24.5 ± 5.0
Direct violence against the neck as cause of death	*21*/*38.9%*
Hanging	8/14.8%
Manual strangulation	5/9.3%
Ligature strangulation	2/3.7%
Sharp force	1/1.9%
Unclear compression of the neck	5/9.3%
Accidents with injury of the neck	*7*/*13.0%*
Fall from < 10 m	1/1.9%
Fall from > 10 m	4/7.4%
Traffic accident	2/3.7%
No evidence of violence against the neck	*26*/*48.1%*

The categorical variables are presented as absolute counts and percentages (*n*/%), and the continuous variables are presented as means and standard deviations (mean ± SD)

*BMI* body mass index

### PMCT

The IQ was compromised by advanced decay (7/13.0%), incisions during autopsy (3/5.6%), and separation of the hyoid from the cartilaginous components (12/22.2%). The IQ of 96.2% of the hyoid bones, 90.7% of the thyroid, and 98.1% of the cricoid cartilages was rated as good or excellent. The hyoid was predominantly analyzed in the bone window, while the majority of the thyroid and the cricoid cartilages were evaluated in both windows (Table 2).

**Table 2:** Image quality and windowing of the three LHC components in PMCT

	Hyoid	Thyroid	Cricoid	*p*
Image quality				
Poor	0/0.0%	0/0.0%	0/0.0%	< 0.001*
Fair	2/3.7%	5/9.3%	1/1.9%	
Good	10/18.5%	21/38.9%	4/7.4%	
Excellent	42/77.8%	28/51.9%	49/90.7%	
Windowing				
Bone	53/98.1%	1/1.9%	2/3.7%	< 0.001*
Soft tissue	0/0.0%	15/27.8%	13/24.1%	
Both	1/1.9%	38/70.4%	39/72.2%	

The categorical variables are presented as absolute counts and percentages (*n*/%)

*The chi-square test was applied to compare the image quality and the windowing of the LHC components in PMCT

PMCT detected a total of 91 fractures in 32 specimens (59.3%). Of these, 23 (25.3%) were located in the hyoid, 50 (54.9%) in the thyroid, and 18 (19.8%) in the cricoid, respectively. The majority of the identified fractures were displaced (47.3%), while fissures were less frequent than non-displaced fractures (20.9% and 31.9%; Table 3).

**Table 3:** Agreement of the LHC fractures in PMCT and PMFP used as the gold standard; the categorical variables are presented as absolute counts and percentages (*n/*%)

	PMCT	PMFP	κ*	*p*
Localization	*91*/*100.0%*	*119*/*100.0%*	*0.762*	*< 0.001*
Hyoid	*23*/*25.3%*	*32*/*26.9%*	*0.818*	*< 0.001*
Corpus	4/4.4%	6/5.0%	0.780	< 0.001
Rostral part of right horn	4/4.4%	6/5.0%	0.780	< 0.001
Occipital part of right horn	5/5.5%	6/5.0%	0.899	< 0.001
Rostral part of left horn	2/2.2%	4/3.4%	0.649	< 0.001
Occipital part of left horn	8/8.8%	10/8.4%	0.867	< 0.001
Thyroid	*50*/*54.9%*	*53*/*44.5%*	*0.831*	*< 0.001*
Centerline	3/3.3%	3/2.5%	1	< 0.001
Right shield	5/5.5%	4/3.4%	0.637	< 0.001
Upper right horn	15/16.5%	16/13.4%	0.864	< 0.001
Lower right horn	1/1.1%	1/0.8%	1	< 0.001
Left shield	4/4.4%	4/3.4%	0.730	< 0.001
Upper left horn	19/20.9%	21/17.6%	0.762	< 0.001
Lower left horn	3/3.3%	4/3.4%	0.847	< 0.001
Cricoid	*18*/*19.8%*	*34*/*28.6%*	*0.568*	*< 0.001*
Rostral centerline	4/4.4%	3/2.5%	0.847	< 0.001
Occipital centerline	3/3.3%	5/4.2%	0.731	< 0.001
Right side	6/6.6%	15/12.6%	0.491	< 0.001
Left side	5/5.5%	11/9.2%	0.427	0.001
Degree of dislocation	*91*/*100.0%*	*119*/*100.0%*	*0.689*	*< 0.001*
Fissure	*19*/*20.9%*	*38*/*31.9%*	*0.295*	*< 0.001*
Hyoid	4/4.4%	7/5.9%	0.351	< 0.001
Thyroid	10/11.0%	7/5.9%	0.339	< 0.001
Cricoid	5/5.5%	24/20.2%	0.247	< 0.001
Non-displaced fracture	*29*/*31.9%*	*31*/*26.1%*	*0.620*	*< 0.001*
Hyoid	4/4.4%	7/5.9%	0.722	< 0.001
Thyroid	14/15.4%	15/12.6%	0.606	< 0.001
Cricoid	11/12.1%	9/7.6%	0.581	< 0.001
Displaced fracture	*43*/*47.3%*	*50*/*42.0%*	*0.830*	*< 0.001*
Hyoid	15/16.5%	18/15.1%	0.839	< 0.001
Thyroid	26/28.6%	31/26.1%	0.829	< 0.001
Cricoid	2/2.2%	1/0.8%	0.664	< 0.001

The values in italics are summaries of the values listed bellow them

*PMCT* post-mortem computed tomography, *PMFP* post-mortem fine preparation

*Cohen’s Kappa (κ) was used to explore the agreement between PMCT and PMFP

PMCT further identified normal variants of the LHC in 46 cases: one case with agenesis of a thyroidal horn, 18 specimens with foramina thyroidea, 21 cartilagines triticeae, and 6 cases with aplasia of both horns of the hyoid.

The inter-reader agreement for the location and the degree of dislocation of the fractures and for the presence of normal variants was strong with *κ* values of 0.89, 0.81, and 0.93 (*p* < 0.001), respectively.

### PMFP

PMFP revealed a total of 119 fractures in 34 specimens (63.0%). Of these, 32 (26.9%) were found in the hyoid, 53 (44.5%) in the thyroid, and 34 (28.6%) in the cricoid. The largest share of the identified fractures was displaced (42.0%), while fissures were more frequent than non-displaced fractures (31.9% and 26.1%).

Direct violence against the neck was the cause of death in 18 of these 34 cases (52.9%), while 7 (20.6%) died from an accident. In the remaining 9 cases (26.5%), there was no suspicion of violence against the neck before autopsy.

PMFP further identified normal variants in 46 cases.

### Comparison of PMCT to PMFP

**Fig. 3:**
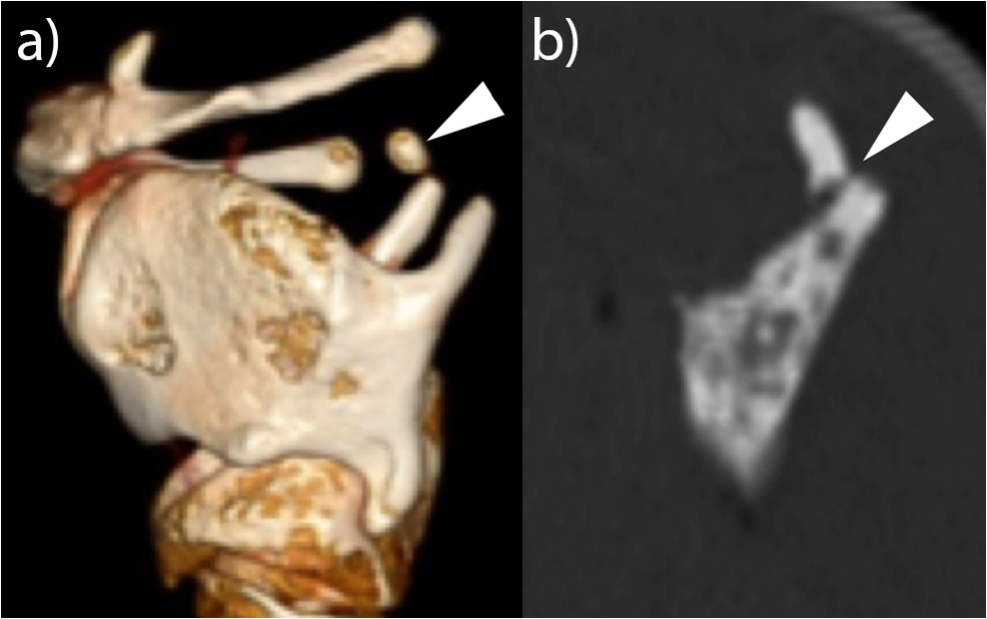
Illustration of the difference between a normal variant and an injury of the isolated LHC in PMCT. **a** Three-dimensional reconstruction of the PMCT scan of an isolated LHC with a right-sided cartilago triticia in its typical position above the upper horn of the thyroid and below the occipital part of the hyoidal horn (lateral view from the left side). **b** PMCT slice of an isolated LHC with a right-sided displaced fracture of the tip of the upper horn of the thyroid reformatted in the bone window (lateral view from the left side)

**Fig. 4:**
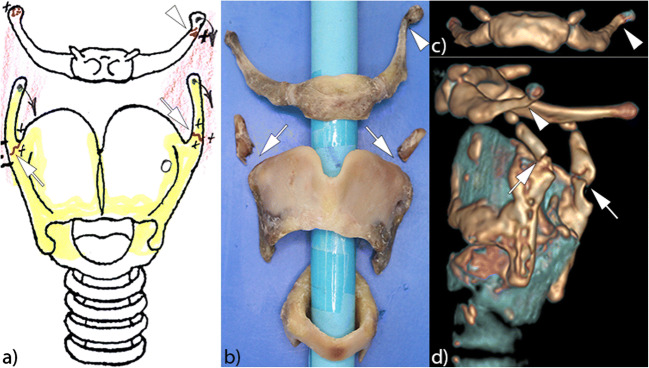
Illustration of an isolated LHC with significantly displaced bilateral fractures of the upper horns of the thyroid (white arrows) and with a tilted fracture of the left occipital horn of the hyoid (white arrowheads). **a** Excerpt of the standardized documentation schema of the PMFP (anterior view). **b** Photograph of the isolated LHC specimen after the PMFP (anterior view). **c** Three-dimensional reconstruction of the PMCT scan of the hyoid (anterior view). **d** Three-dimensional reconstruction of the PMCT scan of the entire LCH complex (lateral view from the left side)

All normal variants detected in PMCT corresponded with PMFPs (Fig. 3). PMCT also significantly agreed with PMFP concerning the localization of the fractures (*κ* = 0.762; *p* < 0.001). The best agreement was achieved for the thyroid (*κ* = 0.831; *p* < 0.001), as PMCT correctly detected all fractures of the centerline and the lower right horn. Concordance for the upper right horn and the lower left horn was good as well (Fig. 4). Good agreements were further attained for the hyoid (*κ* = 0.818; *p* < 0.001), especially for the occipital part of both horns. The poorest match was seen for the cricoid, where fractures of both sides were frequently missed on PMCT (*κ* = 0.568; *p* < 0.001; Table 3).

Concerning the degree of dislocation of the fractures, good agreements of PMCT and PMFP were achieved for both displaced and non-displaced fractures, while fissures were frequently missed on PMCT (Table 3).

PMCT correctly identified 30 injured LHCs, as well as 18 non-injured specimens. PMCT image analysis missed, however, 4 LHCs, which were identified to be injured in PMFP. Similarly, images of 2 LHCs were falsely interpreted as damaged in PMCT, while they were intact in PMFP. Comparing the outcome of PMCT to PMFP resulted in a sensitivity of 88.2%, a specificity of 90.0%, and an accuracy of 88.9%. Separate comparisons of the outcomes of each LHC component resulted in a sensitivity and specificity of 84.2% and 100.0% for the hyoid, of 82.1% and 92.3% for the thyroid, and of 44.4% and 100.0% for the cricoid, respectively. The sensitivity of the cricoid and the entire LHC significantly differed from each other, while the confidence intervals of all other parameters overlapped (Table 4), indicating a lack of significant differences.

**Table 4:** Case-based calculation of the diagnostic accuracy of PMCT compared to PMFP as the gold standard

	PMFP		Sum	SENS (95% CI)	SPEZ (95% CI)	ACC (95% CI)
	Injured	Intact				
LHC						
PMCT						
Injured	30	2	32	88.2% (0.73–0.97)*	90.0% (0.68–0.99)	88.9% (0.77–0.96)
Intact	4	18	22			
Sum	34	20	54			
Hyoid						
PMCT						
Injured	16	0	16	84.2% (0.60–0.97)	100.0% (0.90–1.0)	94.4% (0.85–0.99)
Intact	3	35	38			
Sum	19	35	54			
Thyroid						
PMCT						
Injured	23	2	25	82.1% (0.63–0.94)	92.3% (0.75–0.99)	87.0% (0.75–0.95)
Intact	5	24	29			
Sum	28	26	54			
Cricoid						
PMCT						
Injured	8	0	8	44.4% (0.22–0.69)*	100.0% (0.90–1.0)	81.5% (0.69–0.91)
Intact	10	36	46			
Sum	18	36	54			

*LHC* larynx-hyoid complex, *PMCT* post-mortem computed tomography, *PMFP* post-mortem fine preparation, *SENS* sensitivity, *SPEZ* specificity, *ACC* accuracy, *CI* confidence interval

*The confidence intervals of LHC and cricoid do not overlap. Therefore, it can be concluded that the lower sensitivity for the cricoid is statistically significant

### Technical parameters

The average CTDIvol was 103.8 ± 10.2 mGy, and the mean DLP was 1406.3 ± 167.0.

On average, the PMCT scan of the LHCs took 3.3 ± 0.6 min while the orthograde adjustment of the images and the calculation of the reformations required 12.1 ± 2.1 min. The mean time for image analysis was 13.5 ± 3.5 min. The average duration of the PMFP including its systematic documentation was significantly higher when compared to the entire PMCT procedure (208.2 ± 32.5 min vs. 28.9 ± 4.1 min; *p* < 0.001).

## Discussion

PMCT correctly detected injured LHCs with a sensitivity of 88.2% and a specificity of 90.0%, when compared to PMFP as the gold standard. Separate analysis of the LHC components revealed comparable results for the thyroid and the hyoid, while injuries of both sides of the cricoid were frequently missed on PMCT. Nevertheless, the cricoid achieved the best IQ. The detection rate of dislocated fractures on PMCT was high, while fissures were frequently missed. In contrast to the bony hyoid, PMCT analysis of the cartilaginous components required the use of both, the soft tissue and bone windows. The entire PMCT procedure including scanning, calculation of the reformations, and image analysis was more time efficient than the performance and documentation of PMFP.

In this cohort, injuries of the thyroid were the most frequent; the most commonly affected parts were both upper horns, which is in concordance with literature that describes these fractures as a typical in lethal neck injuries [10, 18–20]. The rather fragile upper horns bend down ventrally when the entire thyroid is pressed against the spine during hanging or strangling, for example [20, 21]. The lower horns are comparatively smaller and are fixed by the cricoarytenoid articulation. Fractures of the shields are common in stabbing and cutting wounds [21]. Comparing PMCT to PMFP, the best detection rate was seen in injuries of the thyroid, particularly fractures of the thyroidal horns, as these are often dislocated and starkly evident when compared to the intact opposite site. Fractures of the less ossified shields were comparatively rare and more difficult to detect even in the soft tissue window, as fissures or non-displaced fractures dominated.

The hyoid was less frequently affected; the occipital parts of the horns were the preferential and best detectable localization, due to the mechanism described above. Left-sided injuries of the hyoid and the thyroid were most prevalent. Previously, Ito et al [22] showed that regardless of gender and age, the left horn of the hyoid is more often fused with the corpus than the right horn. One explanation may be the handedness. As the majority of the population is right-handed, the left side of the jaw is more exposed to the effects of forces from mastication or strong static actions with the non-dominant hand. Therefore, the left side develops a higher grade of ossification and is less flexible and more prone to fractures [22, 23]. This asymmetry, and surface irregularities of the corpus, further impedes the discrimination between rostral fractures and areas of ossification where the corpus and the horns fuse.

In concordance with the literature [8, 20, 21], fractures of the cricoid were infrequent and fissures, which are difficult to detect, dominated. Fractures of the cricoid may be so uncommon due to the stabile ring structure and the inhomogeneous mixture of bony and cartilaginous parts, which provide higher elasticity and absorption of direct forces. This further applies to the shields and lower horns of the thyroid as well as the corpus of the hyoid [21, 24]. Fractures of these more stable elements were comparatively less common in the cohort and may indicate a higher intensity of trauma. Additionally, the degree of ossification influences the stability and a higher degree of ossification causes more vulnerability [23, 25].

So far, several studies have investigated the performance of PMCT in comparison to autopsy for the detection of laryngeal injuries with a broad spectrum of sensitivities, ranging from 53 to 85% [11, 12, 19, 26]. Dissimilar to our study, all former investigations examined the LHC in situ but did not isolate it and scan it with an adequate field of view such that the in-plane resolution increased. Furthermore, PMCT of extracted LHC specimens differs from whole-body scans due to the absence of the cervical spine, which increases the homogeneity of the scan area and eliminates artifacts, thus improving the image quality. Artifacts caused by intubation, elevated arms, or extracorporeal foreign objects are eliminated as well. However, scans of the extracted LHC specimens prevent the evaluation of associated mucosal damage. This can either be advantageous or disadvantageous, as injuries of the surrounding tissue detract from laryngeal damages but also serve as an indicator for associated LHC fractures [21]. Nevertheless, a PMCT scan prior to autopsy is certainly necessary as a reference and, by now, it is practice at our center.

Most former studies compared PMCT to autopsy instead of PMFP. The few studies that used PMFP as standard of reference presented a comparison to findings of X-ray examinations [9, 27], and they imply the major limitation that fissures and non-displaced fractures were undetectable in predominantly or completely cartilaginous LHC components. This primarily applies to the thyroid and the cricoid in specimens of younger individuals. Khokhlov [19] describes the complete preparation and stereomicroscopy of the LHC as the *gold standard* method, which enables the detection of even minimal signs of destruction and avoids false-positive diagnoses of injuries. Additionally, the application of aniline dye during PMFP is the only method, which reliably illuminates the direction of a fracture, which is fundamental to discovering the causal mechanism of injury. The complete preparation, however, is time-consuming and requires practice. In order to avoid additional trauma during autopsy, manipulation should be as minimal as possible.

The investigation of normal variants of the LHC avoided a potential confounder as they can be mistaken for injuries. The frequency of cartilago triticea, which resembles a dislocated fragment, ranges from 13.2 to 53.1% in the literature [10, 28, 29]. The rather high incidence of 38.9% in the cohort is probably due to the ability of PMCT to visualize bony and cartilaginous samples, while observations based on X-rays alone miss these cartilaginous samples. The incidence of foramina thyroidea due to irregular ossification of the thyroidal shields was comparable to the literature (33.3% vs. 28.3%) [16]. An agenesis of the thyroidal horn or an aplasia of the hyoidal horns is comparatively rare but may also imitate dislocated fractures, especially in cases of advanced decay or in victims with deep snags [29].

The first limitation of this study is the rather small sample size, as it was a single center study with strict inclusion criteria. Moreover, there was only one investigator who performed the PMFP and thus no inter-preparator reproducibility can be presented for this specific method. Otherwise, results are reliable due to the fact that one highly experienced person performed all PMFPs in a standardized procedure and that all scans were performed in the same way on the same scanner and at the same institution.

Secondly, the study population was pre-selected, so samples with an obvious cause of death, which had been assessed by autopsy, were excluded. These cases, however, would not normally undergo a time-consuming PMFP in daily routine. Thus, the exclusion of these samples reflects standard practice.

The third limitation of this in vitro study was that both PMFP and PMCT used samples post autopsy, and thus, the results of both methods could be biased by autopsy-related injuries. Moreover, evaluation of the adjacent soft tissue, where hemorrhage is a potent indicator of injuries of the surrounding cartilage, was impossible [21]. On the other hand, the LHCs were retrieved through a standardized process with a high degree of care, so the well-preserved LHCs could be imaged in their plastic containers during a daily routine in a clinical CT scanner.

In conclusion, PMCT of the isolated LHC is more time efficient than PMFP and can detect distinct injuries like fractures of bony components. It may therefore be used to promptly confirm the suspicion of severe violence against the neck as the cause of death in most cases, which may reduce the number of cases and require the more time-consuming process of PMFP. In contrast to PMFP, the stored PMCT images are re-evaluable anytime. PMFP outperforms PMCT, however, in the detection of decent injuries like cartilage fissures. This limits its application in forensic cases with suspicion of slight neck trauma as well as in persons deceased at a young age since their LHC components are predominantly cartilaginous. In comparison to PMFT, however, PMCT also missed some non-displaced and displaced fractures. Therefore, it is important to always keep the limitations of PMCT in mind, and in forensic daily routine it is reasonable to choose the diagnostic path individually according to the specific circumstances of each case.

## Abbreviations

ACC: Accuracy
CTDIvol: Volume-weighted CT dose index
DLP: Dose length product
IQ: Image quality
*κ*: Cohen’s kappa value
LHC: Larynx-hyoid complex
PMCT: Post-mortem computed tomography
PMFP: Post-mortem fine preparation
