# Correction to: Injuries of the isolated larynx-hyoid complex in post-mortem computed tomography (PMCT) and post-mortem fine preparation (PMFP) - a comparison of 54 forensic cases

**DOI:** 10.1007/s00330-021-08135-x

**Published:** 2021-07-16

**Authors:** Karla Maria Treitl, Laura Isabel Aigner, Evgenij Gazov, Florian Fischer, Regina Schinner, Christine Schmid-Tannwald, Sonja Kirchhoff, Michael Karl Scherr

**Affiliations:** 1 Department of Radiology, University Hospital, LMU Munich, Pettenkoferstr. 8a, 80336 Munich, Germany; 2 Department of Radiology, Klinikum Kempten, Kempten, Germany; 3 Department of Radiology, BG Unfallklinik Murnau, Murnau am Staffelsee, Germany; 4 Institute of Forensic Medicine, Ludwig Maximilians University, Munich, Germany

**Correction to: European Radiology (2020) 30:4564–4572**

10.1007/s00330-020-06770-4

The article “Injuries of the isolated larynx-hyoid complex in post-mortem computed tomography (PMCT) and post-mortem fine preparation (PMFP) - a comparison of 54 forensic cases“, written by Treitl, K.M., Aigner, L.I., Gazov, E., Fischer, F., Schinner, R., Schmid-Tannwald, C., Kirchhoff S. and Scherr, M.K., was originally published Online First without Open Access. After publication in volume 30, issue 8, page 4564–4572 the author decided to opt for Open Choice and to make the article an Open Access publication. Therefore, the copyright of the article has been changed to © The Author(s) 2020 and the article is forthwith distributed under the terms of the Creative Commons Attribution 4.0 International License, which permits use, sharing, adaptation, distribution and reproduction in any medium or format, as long as you give appropriate credit to the original author(s) and the source, provide a link to the Creative Commons license, and indicate if changes were made. The images or other third party material in this article are included in the article’s Creative Commons license, unless indicated otherwise in a credit line to the material. If material is not included in the article’s Creative Commons license and your intended use is not permitted by statutory regulation or exceeds the permitted use, you will need to obtain permission directly from the copyright holder. To view a copy of this license, visit http://creativecommons.org/licenses/by/4.0/.

The original article has been corrected.

